# Exploring the gut–brain axis: dietary influences on Alzheimer’s disease pathogenesis

**DOI:** 10.3389/frmbi.2026.1639904

**Published:** 2026-06-08

**Authors:** Samira R. Mansour, Menna A. Khalaf, Mostafa A. Moustafa, Mardies A. Moustafa, AbdelRaouf A. Moustafa

**Affiliations:** 1 Suez Canal University, Faculty of Science, Ismailia, Egypt; 2 Neurosurgey, October 6 University Faculty of Medicine, 6th of October City, Egypt; 3 Faculty of Dentistry, Sinai University, El-Kantra, Egypt

**Keywords:** Alzheimer’s disease, amyloid plaques, dietary factors, dysbiosis, gut barrier integrity, gut microbiome, gut–brain axis, immune response

## Abstract

Alzheimer’s disease (AD) is one of the most diagnosed neurodegenerative disorders worldwide and presents a significant challenge for both affected individuals and their caregivers. Alzheimer’s disease is characterized by the accumulation of amyloid plaques and dysfunctional tau protein in the brain, along with the final development of dementia. Recently, in addition to the strongly developing ischemic etiology of AD, it is suggested that the gut and oral microbiota may also participate in the development of this disease. This involvement may stem from an unbalanced diet and the consumption of foods containing harmful chemical additives. An unhealthy diet can compromise the integrity of the gut barrier, facilitating the translocation of bacterial pathogens and leading to a pro-inflammatory T-cell response mediated by innate immune cells. This inflammatory response can disrupt systemic homeostasis and may contribute to neuroinflammation. The brain and gut interact through a complex network known as the “gut–brain–microbiota axis,” and emerging studies suggest that the intestinal microbiota and their metabolites may play a significant role in the pathogenesis of Alzheimer’s disease. Moreover, these inflammatory mediators and microbial metabolites can reach the brain via the gut–brain axis, potentially exacerbating neurodegenerative processes. Preclinical and limited clinical evidence indicates that low-fiber diets are associated with alterations in intestinal microbiota composition, which may contribute to the onset and progression of Alzheimer’s disease. This review aims to explore the potential connections between AD and the gut microbiome, emphasizing the significance of dietary factors in shaping these relationships. A comprehensive understanding of the interactions between the human microbiome and the brain, particularly in the context of diet and its ingredients, may enhance our understanding of AD etiology and inform the development of preventative strategies, through dietary modifications or therapeutic interventions. This area of research holds promise for identifying novel approaches to prevent or slow the progression of AD.

## 1 Introduction

Alzheimer’s disease (AD) is a progressive neurodegenerative disorder characterized by cognitive decline, memory loss, and behavioral changes, affecting millions of aged individuals worldwide. It is an irreversible neurodegenerative process that progressively impairs brain function, causing disruptions in memory, cognition, personality, and other functions that interfere with everyday activities and eventually lead to death from complete brain failure. AD has emerged as one of the most lethal, economically burdensome, and socially impactful diseases of the 21st century. It has a major impact on both individuals who suffer from it and the healthcare system, as well as increasing the burden on caregivers. The exact cause of AD remains unclear but is attributed to a combination of genetic, environmental, and lifestyle factors influencing its onset and progression (Hickman et al., 2024). Age is the predominant risk factor, with the incidence nearly doubling every 5 years after 65 and markedly increasing by age 85 (Wang et al., 2025), highlighting its critical role in AD epidemiology. Currently, approximately 50 million people worldwide have AD, with projections estimating that this number will triple to 152 million by 2050 (WHO, 2025; Breijyeh and Karaman, 2020; Nichols et al., 2019).

According to the Alzheimer’s Association (2021), the prevalence of AD escalates with advancing age. Specifically, approximately 5% of individuals aged 65 to 74 are affected by AD. This figure rises to approximately 13% for those aged 75 to 84 and significantly increases to approximately 33% for individuals aged 85 and older (Alzheimer’s Association, 2021; Fang et al., 2025). These statistics highlight a crucial aspect of Alzheimer’s epidemiology: the older a person is, the higher their risk of developing the disease. Despite extensive research, the exact mechanisms underlying the onset and progression of AD remain mysterious, posing a significant challenge for effective treatment and prevention.

**Figure 1:**
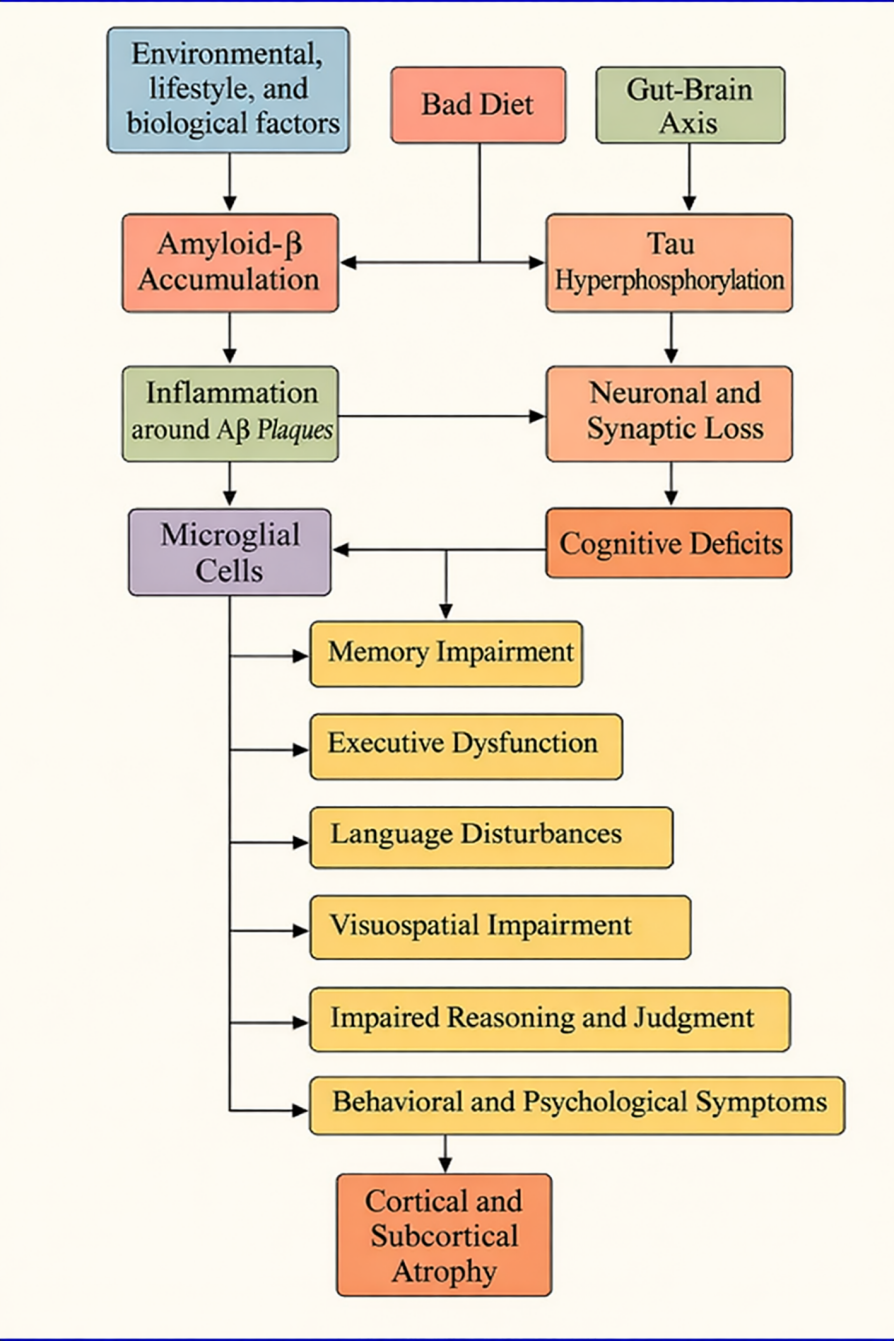
Scientific flowchart diagram illustrates the pathogenic cascade, linking dietary factors in addition to environmental, lifestyle, and biological factors, that contribute to the development and progression of Alzheimer’s disease. These factors induce gut microbiota dysbiosis, resulting in increased intestinal permeability and systemic inflammation via the gut–brain axis. These peripheral inflammatory signals facilitate amyloid-β aggregation and tau protein hyperphosphorylation within the central nervous system. These changes trigger neuroinflammation and microglial activation, leading to neuronal and synaptic loss. As the damage progresses, cognitive deficits emerge, ultimately resulting in cortical and subcortical brain atrophy.

AD pathology is characterized by extracellular beta-amyloid (Aβ) peptide accumulation, intracellular hyperphosphorylated tau-containing neurofibrillary tangles, neuroinflammatory mediator deposition, and neuronal and synaptic loss in the central nervous system. These interconnected events lead to the cognitive deficits typical of AD and result in cortical and subcortical atrophy (**Figure 1**). Environmental, lifestyle, and biological factors, including poor dietary habits via the gut–brain axis, may contribute to Aβ accumulation and tau hyperphosphorylation. These changes trigger neuroinflammatory responses with microglial activation around amyloid plaques, which exacerbate neuronal and synaptic damage. While the brain’s innate immune system maintains central nervous system (CNS) integrity, chronic microglial activation is neurotoxic and significantly influences AD progression. Thus, inflammation is considered both a consequence and a major driver of the disease (**Figure 1**).

## 2 Role of amyloid-β accumulation and neuroinflammation

**Figure 2:**
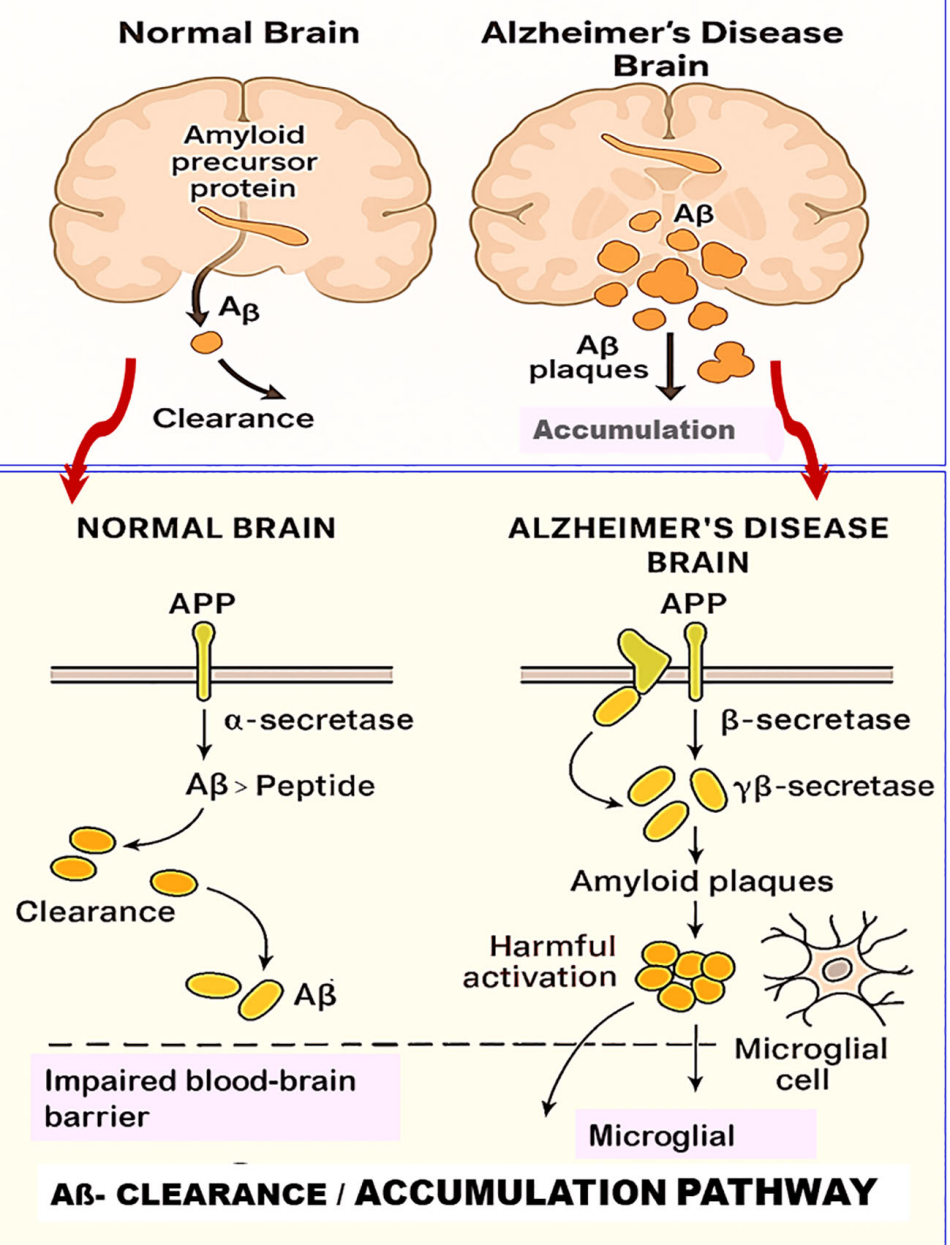
This figure highlights the balance between Aβ production and clearance. Amyloid-β (Aβ) clearance vs. accumulation in normal and Alzheimer’s disease brains. **(A)** In a normal brain, amyloid precursor protein (APP) undergoes non-amyloidogenic processing, and any Aβ generated is effectively cleared from the brain, maintaining homeostasis. In contrast, in an Alzheimer’s disease brain, Aβ peptides are overproduced and accumulate, forming toxic Aβ plaques that are not efficiently cleared. **(B)** The APP molecular pathway in which it is cleaved by α-secretase, a non-amyloidogenic pathway that generates soluble peptides. Any Aβ produced is cleared across the blood–brain barrier. In the AD brain, APP is abnormally processed by β-secretase and γ-secretase, generating excessive Aβ peptides that aggregate into amyloid plaques. This accumulation leads to impaired clearance, partly due to a compromised blood–brain barrier. Harmful activation of microglial cells, the brain’s immune cells, contribute to neuroinflammation and further neuronal damage. This figure highlights the critical balance between Aβ production and clearance, and how its dysregulation underlies Alzheimer’s disease pathology.

Amyloid-beta (Aβ) is a peptide generated through the sequential enzymatic cleavage by α-, β-, and γ-secretases of amyloid precursor protein (APP), a transmembrane protein predominantly expressed in neurons. Under normal physiological conditions, Aβ is produced at low levels and is effectively cleared from the brain via enzymatic degradation and transport across the blood–brain barrier. In a study done by Waly et al. (2023), a decline in neuronal plasticity associated with aging was observed, which is further exacerbated inan *in vivo* Alzheimer’s disease model and correlates with cognitive decline. In Alzheimer’s disease, however, there is an imbalance between Aβ production and clearance, resulting in its pathological accumulation (Huang et al., 2025a). As illustrated in **Figure 2**, the processing of APP differs between healthy and AD brains. Under normal physiological conditions, APP is predominantly cleaved by α-secretase, producing non-amyloidogenic peptides that are efficiently cleared. In contrast, in AD, APP is sequentially cleaved by β- and γ-secretases, generating Aβ peptides. These peptides undergo a progressive aggregation process, forming soluble oligomers and protofibrils that eventually assemble into insoluble extracellular amyloid plaques (Perneczky et al., 2024). The accumulation of these plaques is a defining neuropathological feature of AD and is implicated in synaptic dysfunction and neurodegeneration.

These Aβ aggregates exert neurotoxic effects through multiple mechanisms. They disrupt synaptic function by interfering with neurotransmitter release and receptor signaling, leading to impaired neuronal communication (Tang et al., 2025). Additionally, Aβ induces oxidative stress by generating reactive oxygen species (ROS), which damage cellular components including lipids, proteins, and DNA. Importantly, Aβ accumulation triggers chronic neuroinflammatory responses mediated by microglia and astrocytes. Microglial cells, the brain’s resident immune cells, initially respond to Aβ deposition by attempting to phagocytose and clear these aggregates (Cohen and Villemagne, 2025). However, persistent exposure to Aβ leads to microglial overactivation, resulting in the sustained release of pro-inflammatory cytokines, chemokines, and other neurotoxic mediators. This chronic inflammatory milieu exacerbates neuronal injury and contributes to synaptic loss.

In patients with AD, neuroinflammation is particularly prominent surrounding amyloid plaques, underscoring the critical involvement of neuroimmune mechanisms in disease progression. The interplay between Aβ pathology and neuroinflammation creates a vicious cycle that amplifies neuronal damage (Zhang and Jiang, 2015; Jorfi et al., 2023). The accumulation of Aβ is now widely recognized as a key initiating event in the cascade of AD pathology, precipitating downstream processes such as tau protein hyperphosphorylation and neurofibrillary tangle formation. These tau-related changes further disrupt neuronal cytoskeletal integrity and intracellular transport, leading to synaptic dysfunction and neuronal death (Aranda-Abreu et al., 2025). Collectively, these pathological events culminate in the progressive cognitive decline characteristic of AD (**Figure 2**).

In murine models, microglia, the resident immune cells of the central nervous system, have been demonstrated to mediate the clearance of Aβ primarily through processes of phagocytosis and lysosomal degradation. Recent studies indicate that microglia actively internalize Aβ aggregates, thereby facilitating a reduction in amyloid plaque burden in AD mouse models (van Olst et al., 2025). This clearance capacity is modulated by both intrinsic microglial activation states and extrinsic factors, such as the gut microbiota, which influence microglial phenotype and phagocytic efficiency (Wasén et al., 2024). In human AD brains, spatial transcriptomic analyses reveal microglia exhibiting distinct transcriptional profiles that cluster around Aβ plaques, indicative of an activated neuroinflammatory state that contributes to both amyloid clearance and disease progression (van Olst et al., 2025). Foundational research also supports the role of microglia in Aβ uptake and degradation, mediated by specific receptors and enzymatic pathways, alongside associated neuroinflammatory responses observed in AD pathology (Lee and Landreth, 2010). Collectively, these findings underscore the dual role of microglia in amyloid pathology: participating in the clearance of Aβ while potentially exacerbating neuroinflammation.

In Alzheimer’s disease brains, inflammation is particularly prevalent around Aβ plaques (Wang et al., 2023; Sun et al., 2024). The initiation of Aβ accumulation and brain inflammation in sporadic AD, where no mutant genes are involved, is not fully understood. In mouse models, microglia, the brain’s resident immune cells, participates in Aβ clearance through phagocytosis and degradation. However, in human sporadic AD, microglia often exhibit dysfunctional or overactivated phenotypes. Chronic inflammation surrounding Aβ plaques suggests an engaged but potentially ineffective or harmful immune response (Liu et al., 2024). The precise triggers for Aβ accumulation and neuroinflammation remain unclear but likely involve aging-related declines in clearance mechanisms, impaired blood–brain barrier function, environmental factors, and systemic metabolic or immune dysregulation (Devkota et al., 2025). These factors contribute to Aβ buildup and sustained neuroinflammatory responses that drive neurodegeneration in AD (Yan et al., 2024).

In an attempt to find a way to recover or at least slow down the progression of Alzheimer’s disease, recent single-nucleus RNA sequencing studies (da Rocha et al., 2025; Wang et al., 2025) highlight the cellular and molecular plasticity underlying this disease. Their work reveals that exercise induces protective transcriptomic changes within specific brain cell populations in the hippocampal dentate gyrus, a critical region for memory formation that is vulnerable early in Alzheimer’s pathology. These adaptive responses include restoration of dysregulated gene expression in immature neurons, activation of neurovascular-associated astrocytes, and modulation of disease-associated microglia and oligodendrocyte precursor cells. Furthermore, the study validated these findings in human Alzheimer’s brain tissue, emphasizing the translational relevance of exercise as a modulator of disease progression.

## 3 Gut microbiome and Alzheimer’s disease development

**Figure 3:**
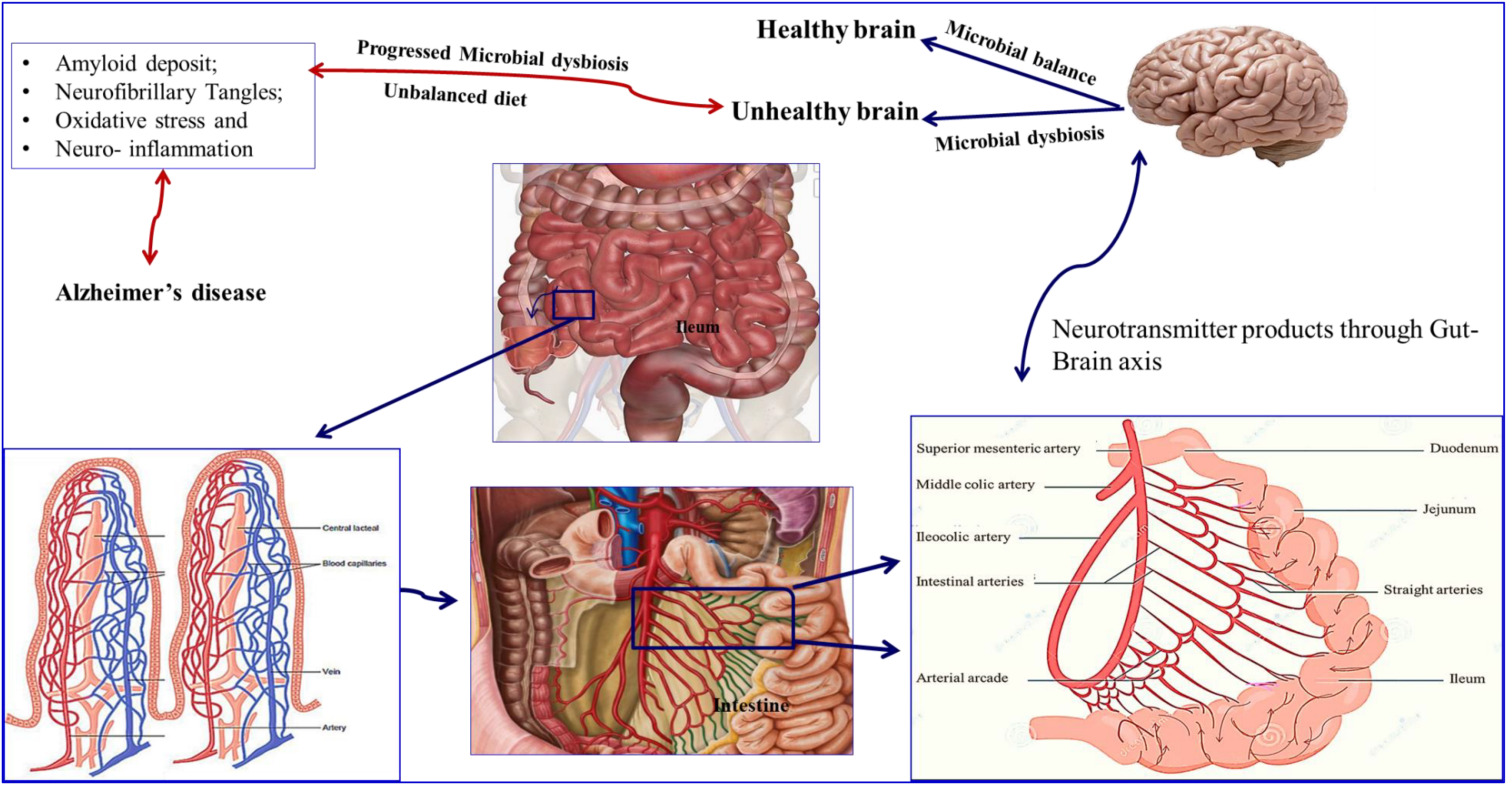
The gut–brain axis is a bidirectional communication system linking the gastrointestinal tract and brain. It involves neural pathways, especially the vagus nerve, and chemical signals like nutrients, microbial metabolites, hormones, and immune molecules. These signals travel through the bloodstream and nerves, influencing brain functions such as mood, cognition, and stress response. This dynamic network maintains physiological balance and coordinates gut and brain activities. At the same time, nerves, especially the vagus nerve, send direct messages from the gut to the brain, reporting on digestion, gut movement, and microbial balance. Together, these pathways help regulate body functions like inflammation, stress response, and emotional behavior, maintaining overall physiological balance (homeostasis).

The human body is colonized by trillions of different microorganisms, collectively known as the microbiome. Microorganisms are considered as accessory organs of the human body, and the microbial communities gathered in different parts of the body to form a mutually beneficial symbiotic relationship with their host (Saad El-Den et al., 2024; Leser and Molbak, 2009). The gastrointestinal tract and the oral cavity are the main distribution sites of symbiotic microorganisms in the human body (Ogobuiro et al., 2020), and several studies have assessed the influence of oral microbiota on AD. In recent years, preclinical and limited clinical evidence has highlighted the gut microbiome as a critical factor influencing the pathogenesis of Alzheimer’s disease. The gut microbiome is a complex ecosystem comprising trillions of microorganisms, including bacteria, archaea, fungi, viruses, and protozoa, and their collective genomes residing primarily in the human gastrointestinal (GI) tract. This microbial community plays essential roles in physiological processes such as digestion, metabolism, immune regulation, and neural communication via the gut–brain axis, a bidirectional network linking the GI tract and the central nervous system (**Figure 3**).

### 3.1 Microbial dysbiosis and intestinal permeability

Microbial dysbiosis refers to an imbalance in the gut microbial community that disrupts normal intestinal functions. This imbalance can increase intestinal permeability, often termed “leaky gut,” allowing microbial products and toxins to cross the gut barrier and trigger systemic inflammation, which has been implicated in various diseases including neurodegenerative disorders. Microbial dysbiosis refers to an imbalance in the gut microbiota, which can disrupt normal intestinal functions. Primarily, studies indicate that alterations in the composition and function of the gut microbiome, a condition termed dysbiosis, contribute significantly to the development and progression of AD, as well as other chronic diseases. In parallel, some studies explore the role of microbial diversity in maintaining gastrointestinal and brain health, emphasizing probiotics and dietary interventions that may alleviate AD-related neuroinflammation and cognitive impairment (Jamerlan et al., 2025; Siripaopradit et al., 2024). They found that probiotic supplementation significantly improved cognitive functions and memory performance in AD animal models which can be explained by the fact that probiotics can modulate gut microbiota composition, reducing dysbiosis, and subsequently decrease neuroinflammation and oxidative stress in the brain.

### 3.2 Blood–brain barrier dysfunction, neuroinflammatory signaling, and microglial activation

**Figure 4:**
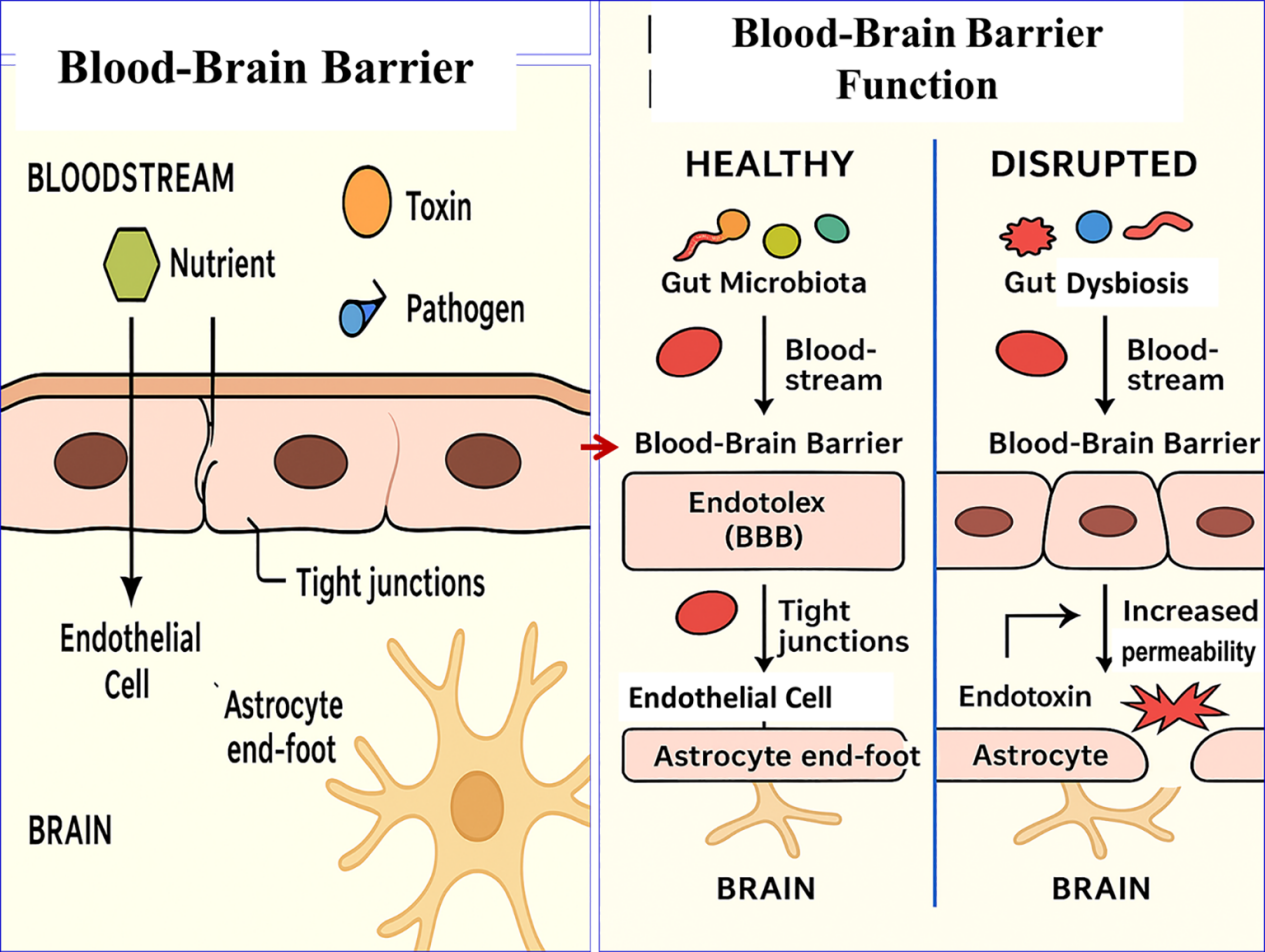
Blood–brain barrier (BBB) integrity in health and Alzheimer’s disease. Under healthy conditions, the BBB tightly regulates brain access, allowing nutrients while blocking toxins and pathogens via tightly joined endothelial cells and astrocyte support. In Alzheimer’s disease, gut microbiota dysbiosis increases intestinal permeability and systemic endotoxin levels, promoting inflammation and weakening tight junctions. This disruption increases BBB permeability, enabling harmful substances to enter the brain and trigger neuroinflammation and disease progression.

The structural integrity of the blood–brain barrier (BBB) regulates the entry of nutrients and protects against toxins and pathogens. Under healthy conditions, the BBB consists of tightly sealed endothelial cells connected by tight junctions, supported by astrocyte end-feet. This structure strictly regulates the passage of substances from the bloodstream into the brain, allowing essential nutrients while preventing the entry of toxins and pathogens. The BBB maintains central nervous system (CNS) homeostasis and protects neural tissue from peripheral immune activation. In Alzheimer’s disease condition, systemic factors such as gut microbiota dysbiosis contribute to BBB disruption (**Figure 4**).

Dysbiosis is characterized by reduced microbial diversity and an increased abundance of pro-inflammatory taxa, such as *Escherichia* and *Shigella* species, which disrupt intestinal homeostasis and promote systemic inflammation (Chen et al., 2024). The gut microbiome regulates host physiology through metabolic, immune, and neuroendocrine signaling pathways. Dysbiosis increases gut permeability, allowing endotoxins to enter the bloodstream. These endotoxins promote systemic inflammation and impair BBB integrity by weakening tight junctions between endothelial cells. This leads to increased permeability, allowing harmful substances to cross into the brain parenchyma, triggering microglial activation and neuroinflammation, and contributing to AD pathogenesis.

Dysbiosis compromises intestinal barrier integrity, increasing permeability to microbial products such as lipopolysaccharides (LPS). These molecules activate systemic inflammatory pathways via Toll-like receptor 4 (TLR4) and nuclear factor kappa B (NF-κB) signaling (Vogt et al., 2018). The resulting inflammatory mediators impair blood–brain barrier function (**Figure 4**), facilitating the entry of peripheral cytokines and gut-derived metabolites, including trimethylamine N-oxide, into the brain. These factors promote neuroinflammation, Aβ(Aβ) aggregation, and tau hyperphosphorylation, key pathological features of AD (Sun et al., 2024). Notably, certain gut bacteria, such as *Bacteroides fragilis*, produce amyloidogenic peptides capable of cross-seeding Aβ plaque formation. Conversely, beneficial genera like *Bifidobacterium* and *Faecalibacterium*, which generate anti-inflammatory short-chain fatty acids (SCFAs), are depleted in AD patients, further exacerbating neuroinflammatory processes (Chen et al., 2024; Haran et al., 2019).

Dysbiosis contributes to AD pathogenesis through several mechanistic pathways:

1. It impairs Aβ clearance by reducing butyrate-dependent upregulation of blood–brain barrier transporters, including low-density lipoprotein receptor-related protein 1 (LRP1) and ATP-binding cassette subfamily B member 1 (ABCB1) (Wang et al., 2025).
2. It induces microglial priming via LPS-mediated inflammasome activation, amplifying neuroinflammatory responses to Aβ and tau pathologies (Ising and Haneka, 2023).
3. It disrupts tryptophan metabolism, shifting synthesis toward the neurotoxic metabolite quinolinic acid and away from serotonin, thereby exacerbating synaptic dysfunction (Savonjie and Weaver, 2023).

Recent interventional studies support the therapeutic potential of targeting the gut microbiome in AD. For example, fecal microbiota transplantation (FMT) from healthy donors reduced Aβ plaque burden and improved cognitive function in AD mouse models by restoring levels of *Akkermansia muciniphila* (Kim et al., 2024; Łysiak and Łysiak, 2024). Similarly, probiotic supplementation with *Lactobacillus* strains decreased cerebrospinal fluid phosphorylated tau (p-tau181) concentrations in early-stage AD patients, suggesting modulation of disease biomarkers (Leblhuber et al., 2023).

These endotoxins, particularly LPS, components of the outer membrane of Gram-negative bacteria, are released into circulation when gut permeability increases due to microbial dysbiosis. Once in the bloodstream, LPS binds to TLR4 on peripheral immune cells such as monocytes and macrophages, leading to the production of pro-inflammatory cytokines like interleukin-1β (IL-1β), interleukin-6 (IL-6), and tumor necrosis factor-alpha (TNF-α). These cytokines circulate systemically and can signal across the blood–brain barrier via several pathways:

1. Disruption of the BBB: Chronic systemic inflammation can compromise the integrity of the BBB, making it more permeable and allowing LPS and immune cells to directly enter the brain parenchyma.
2. Cytokine signaling across the BBB: Even without direct entry, cytokines produced in response to LPS can signal through the circumventricular organs (areas of the brain where the BBB is naturally more permeable) or bind to endothelial receptors, triggering secondary messengers and inflammatory cascades in the CNS. Activation of brain-resident microglia: These signals stimulate microglia, the CNS’s innate immune cells, shifting them toward a pro-inflammatory (M1) phenotype, which promotes neuroinflammation and contributes to Aβ aggregation and neuronal damage.

**Figure 5:**
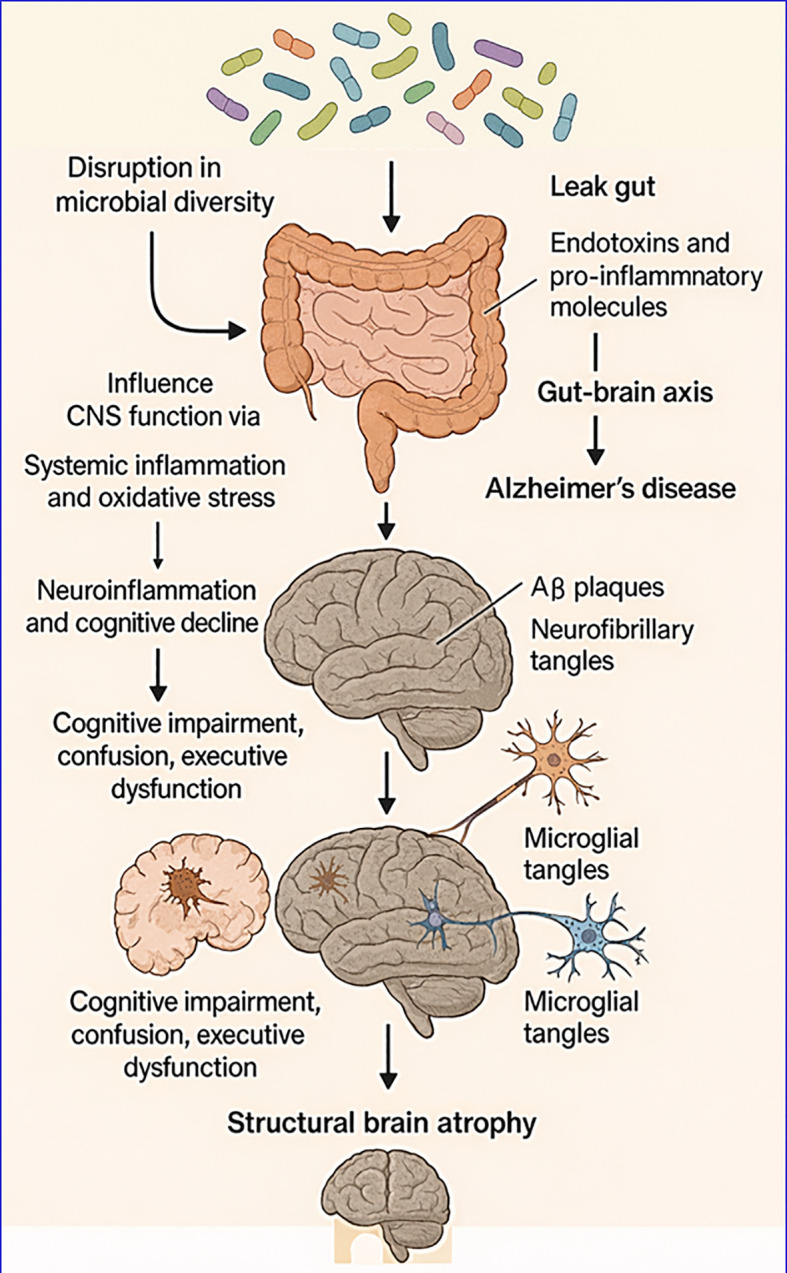
The figure highlights the effect of microbiome dysbiosis and its contributory role in Alzheimer’s disease pathogenesis. The diagram illustrates the proposed pathological cascade linking gut microbiome dysbiosis to the beginning and progression of AD. Altered microbial diversity increases intestinal permeability (“leaky gut”), allowing endotoxins and pro-inflammatory molecules to enter systemic circulation. These factors trigger systemic inflammation and oxidative stress, which influence central nervous system (CNS) function via the gut–brain axis. This disruption promotes neuroinflammation and cognitive decline, accompanied by the accumulation of amyloid-β (Aβ) plaques and neurofibrillary tangles in the brain and microglial activation, collectively driving neuronal damage and cognitive decline in AD.

In conclusion, pathophysiological mechanisms underlying Alzheimer’s disease and the role of diet and other factors lead to severe inflammation and neurodegeneration. These processes can be summarized as follows: an unhealthy diet adversely affects the gut microbiota, leading to dysbiosis and increased intestinal permeability. This disruption allows endotoxins and pro-inflammatory molecules to translocate into the systemic circulation, thereby influencing CNS function via the gut–brain axis. Disruption of the gut–brain axis promotes neuroinflammation and cognitive decline. In the brain, accumulation of misfolded Aβ proteins forms plaques that induce local inflammatory responses, attracting immune cells such as microglia (**Figure 5**). Chronic microglial activation shifts these cells from protective to pro-inflammatory phenotypes, releasing cytokines and reactive oxygen species that damage neurons. Concurrently, abnormal tau hyperphosphorylation leads to the formation of neurofibrillary tangles, disrupting neuronal transport and function. The combined effects of amyloid and tau pathology, along with sustained neuroinflammation, result in widespread neuronal and synaptic loss, manifesting as cognitive deficits including memory impairment, confusion, and executive dysfunction. Structural brain atrophy, particularly in cortical and subcortical regions, characterizes advanced AD, reflecting the culmination of these neurodegenerative processes.

Disruption in microbial diversity: External factors such as aging, diet, antibiotics, and stress alter the composition and diversity of the gut microbiota, reducing beneficial microbes and allowing overgrowth of pro-inflammatory species.

1. Leaky gut and systemic inflammation: Dysbiosis impairs intestinal barrier integrity, leading to increased gut permeability (“leaky gut”). This allows translocation of endotoxins and pro-inflammatory molecules such as LPS into systemic circulation.
2. Gut–brain axis and CNS involvement: Circulating inflammatory molecules influence CNS function via the gut–brain axis, which includes neural (vagus nerve), immune (cytokines), and endocrine pathways. This interaction promotes systemic inflammation and oxidative stress.
3. Neuroinflammation and cognitive decline: Chronic systemic inflammation activates microglia in the brain, shifting them from a protective to a pro-inflammatory phenotype. This results in sustained release of cytokines and reactive oxygen species, contributing to neuroinflammation and early cognitive impairment.
4. Aβ and tau pathology: In the AD brain, misfolded Aβ accumulates into plaques, while tau proteins undergo hyperphosphorylation, forming neurofibrillary tangles. These pathological features disrupt neuronal signaling, synaptic function, and cytoskeletal stability.

## 4 Pathways linking gut microbiome alterations to Alzheimer’s disease development

**Figure 5** illustrates the sequential steps by which changes in the gut microbiome contribute to the development of AD. It highlights the complex interactions connecting microbial dysbiosis, disrupted gut barrier integrity, systemic inflammation, and neuroinflammation that collectively drive AD pathogenesis. This figure provides a visual framework to understand how alterations in gut microbial composition and their metabolites influence brain health and disease progression:

1. Disruption in microbial diversity: An imbalance in gut microbiota reduces diversity, leading to dysbiosis.
2. Increased gut permeability: Dysbiosis causes “leaky gut,” allowing pro-inflammatory molecules to enter the bloodstream.
3. Influence on the CNS: These inflammatory molecules impact CNS function through the gut–brain axis.
4. Systemic inflammation and oxidative stress: Chronic inflammation and oxidative stress occur, contributing to neuroinflammation.
5. Neuroinflammation: This inflammation is associated with the accumulation of Aβ plaques and neurofibrillary tangles in the brain.
6. Cognitive impairment: The presence of Aβ plaques and tangles is linked to cognitive impairment, confusion, and executive dysfunction.
7. Structural brain atrophy: Over time, these changes result in structural brain atrophy, further exacerbating cognitive decline.

## 5 Etiology and consequences of gut microbiome dysbiosis

**Figure 6:**
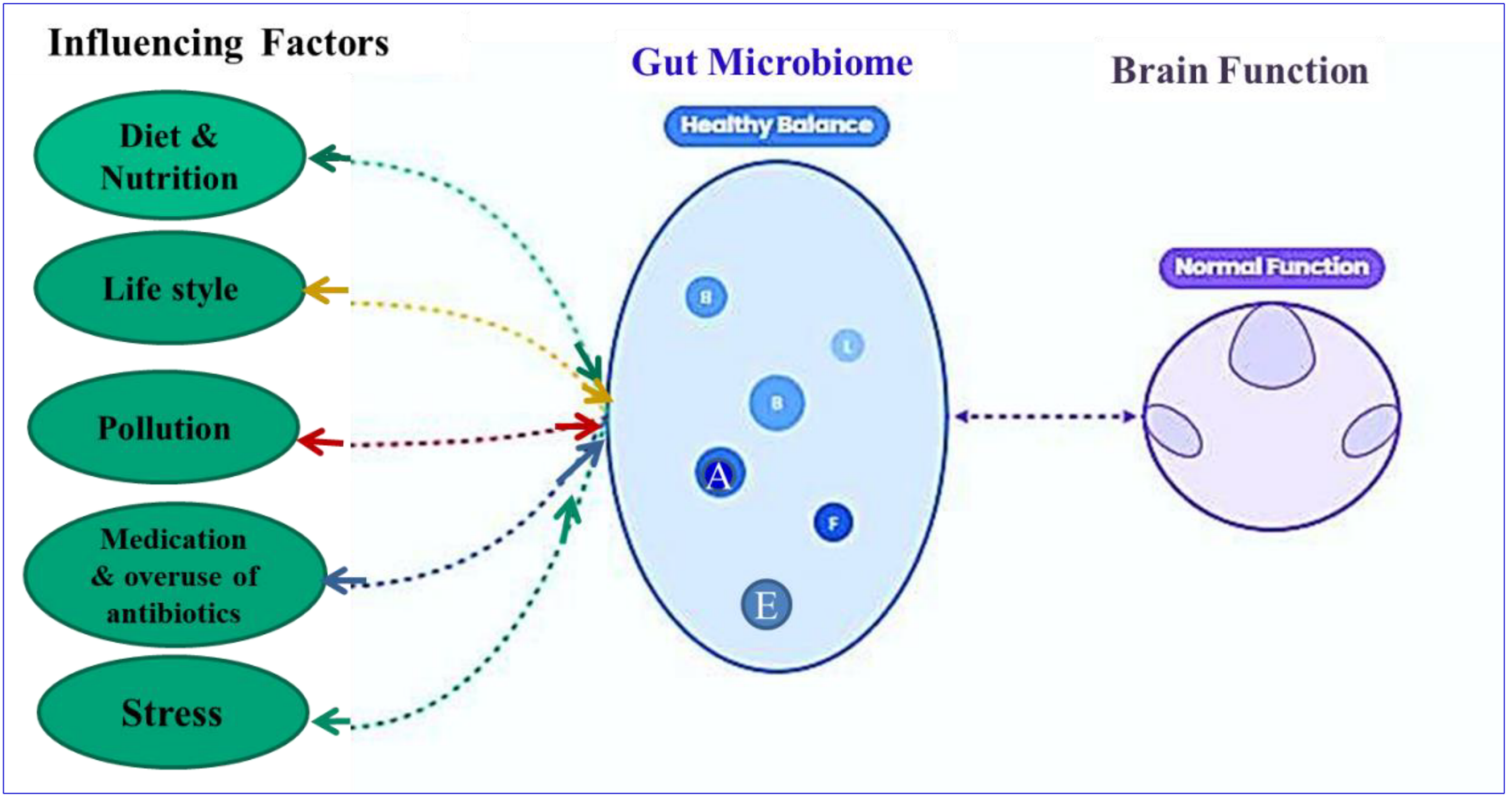
The interconnection between Gut Microbiome, Brain Health, and Etiology Factors. This diagram illustrates various influencing factors, diet and nutrition, lifestyle, pollution, medication and overuse of antibiotics, and stress, affect the gut microbiome and, consequently, brain function. A healthy and balanced gut microbiome, composed of beneficial bacterial groups [represented by **(A–E)**], plays a critical role in maintaining normal brain function via the gut-brain axis. Positive influences such as healthy diet (green arrow) and regular physical activity(Yellow arrow) support microbial diversity and stability, promoting mental well-being. In contrast, negative influences like environmental pollutants (red arrow), chronic stress (greenish blue arrow), and excessive or inappropriate use of antibiotics (blue arrow) can disrupt microbial balance (dysbiosis), potentially impairing brain health through increased inflammation, reduced neurochemical signaling, and altered gut permeability. The figure emphasizes the interconnectedness of lifestyle and environmental factors with gut and neurological health.

Gut microbiome dysbiosis, characterized by reduced microbial diversity, depletion of beneficial commensals, and the overgrowth of opportunistic pathogens, can result from various factors, including environmental exposures, chronic stress, medications (particularly antibiotics), and notably, long-term dietary habits (Aguirre de Carcer, 2025; Saad El-Den et al., 2024). The gut microbiome, composed of a dynamic consortium of bacteria, archaea, fungi, viruses, and their collective genomes residing in the gastrointestinal tract, is highly dynamic and responsive to these external influences (**Figure 6**).

A healthy and balanced gut microbiome contributes to maintaining normal brain function, likely through the gut–brain axis involving neural, hormonal, and immune pathways. Disruption of this balance, known as dysbiosis, results from these factors indiscriminately affecting both beneficial and harmful microbes, leading to decreased microbial diversity and the proliferation of opportunistic pathogens. Such alterations in the gut microbiome can trigger a cascade of effects that extend beyond the gastrointestinal tract, influencing brain function through the gut–brain axis. These consequences may impair neurological health and cognitive function. Bidirectional communication via the gut–brain axis enables continuous two-way signaling between the CNS and the gastrointestinal tract. This complex connection involves neural (vagus nerve), endocrine, and immune pathways, explaining how external factors such as stress can influence digestive processes and how gut health, in turn, can modulate mood, cognition, and overall brain function.

Dysbiosis disrupts the homeostatic functions of the gut microbiota, leading to impaired metabolic activity and altered immune signaling. One key consequence is the compromise of intestinal barrier integrity, often referred to as a “leaky gut,” which increases permeability to microbial-derived components such as LPS. These pro-inflammatory molecules can translocate into systemic circulation, activating immune responses and promoting low-grade chronic inflammation. In the meantime, a healthy and balanced gut microbiome, composed of beneficial bacterial groups, like *Bifidobacterium*, *Lactobacillus*, and *Faecalibacterium*, plays a critical role in maintaining normal brain function via the gut–brain axis (Zhang et al., 2025).

Through the gut–brain axis, a complex bidirectional communication system involving neural (vagus nerve), endocrine (cortisol, gut hormones), and immune (cytokines) pathways, these inflammatory signals and microbial metabolites can influence CNS function (Park and Gao, 2024). Dysbiosis-induced inflammation has been linked to neuroinflammatory processes, altered neurotransmitter production, and impaired cognitive function. In contrast, a balanced and diverse gut microbiome supports brain health by reinforcing intestinal barrier function, regulating peripheral and central immune responses, and synthesizing neuroactive compounds such as SCFAs, tryptophan metabolites (Savonjie and Weaver, 2023), and gamma-aminobutyric acid (GABA).

Recent studies demonstrate that gut microbiota modulate dietary tryptophan metabolism, producing bioactive metabolites such as indole derivatives, kynurenines, and serotonin that influence neurophysiology, mood, cognition, and the gut–brain axis (Yuxuan et al., 2026; Qin et al., 2025) and regulate central nervous system function or impact peripheral pathways with downstream brain effects. Gut microbes achieve this by synthesizing metabolites or altering their availability, underscoring tryptophan’s essential role as a precursor for neuroactive kynurenine pathway products, serotonin, GABA, and indoles. Maintaining microbial homeostasis is therefore critical for preserving neuroimmune balance and mitigating the risk of neurodegenerative diseases such as Alzheimer’s disease.

## 6 Diet-induced microbial dysbiosis and its role in Alzheimer’s disease progression

Regarding diet-induced microbial dysbiosis, nutrients act as building blocks and regulators in metabolism, contributing to energy production, cellular function, and the regulation of gene expression. These nutrients come from either a healthy or an unhealthy diet. Diets high in unhealthy fats, sugars, and processed foods can reduce microbial diversity and promote the proliferation of harmful microbes, particularly bacteria. This imbalance not only disrupts normal gut function but also triggers systemic inflammation, which has been linked to various neurodegenerative diseases, including AD. The altered gut microbiome can produce neurotoxic metabolites that may exacerbate cognitive decline, creating a vicious cycle of worsening health. As Alzheimer’s disease progresses, the impact of microbial dysbiosis becomes increasingly evident. The inflammatory responses initiated by an imbalanced gut microbiome can contribute to the accumulation of amyloid plaques and tau tangles in the brain, hallmark features of Alzheimer’s pathology. Furthermore, the gut–brain axis plays a crucial role in this relationship, as signals from the gut can influence brain health and function. Thus, addressing dietary habits to restore microbial balance may offer a potential therapeutic avenue to slow the progression of Alzheimer’s disease and improve overall cognitive health.

**Figure 7:**
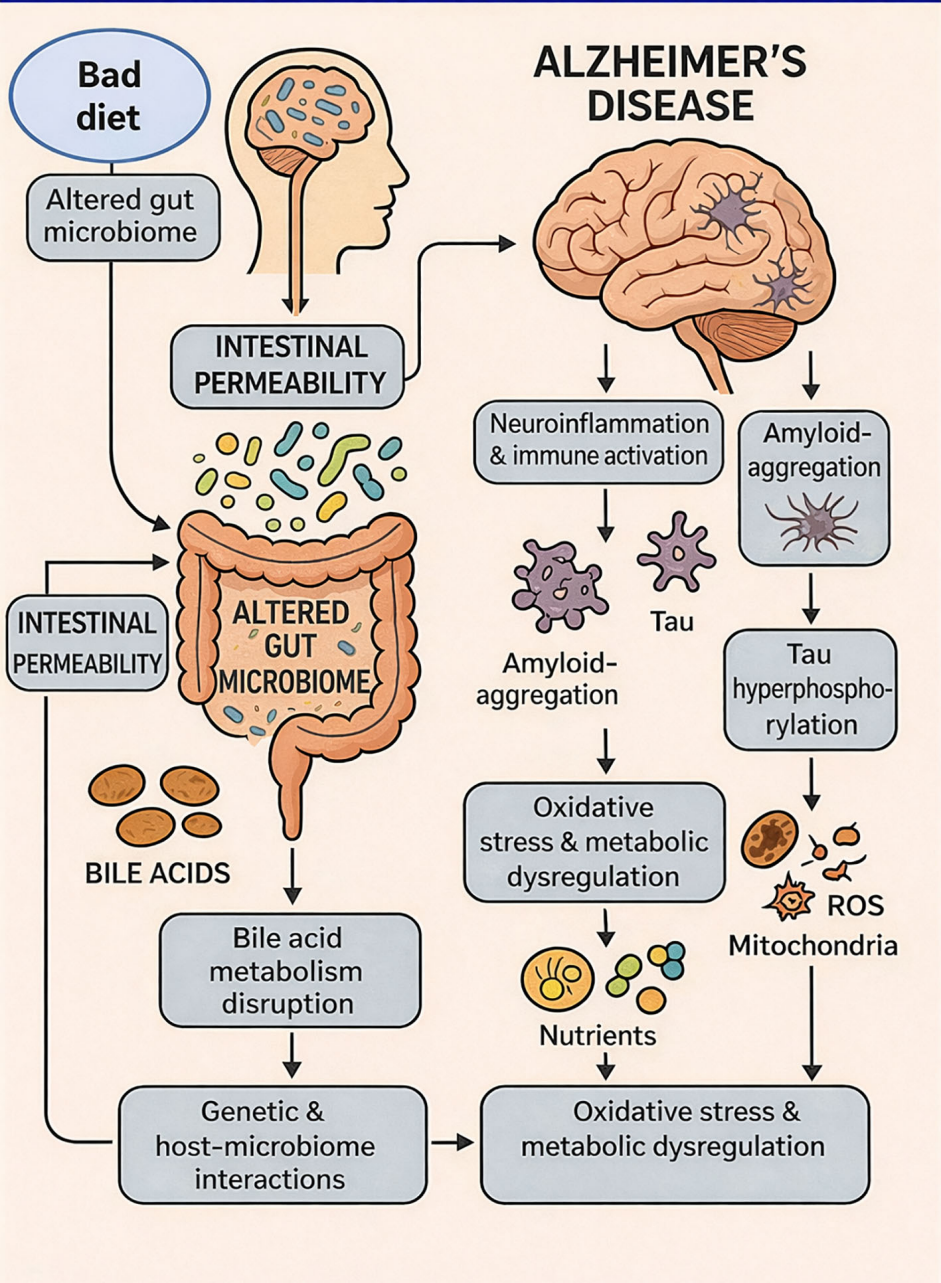
Relation between diet quality and Alzheimer’s disease development: Various mechanisms illustrate the complex interplay between gut health and neurodegenerative processes in Alzheimer’s disease.

The altered gut microbiome in Alzheimer’s disease can contribute to the development and progression of the disease through several interconnected mechanisms: Firstly, the dysbiotic microbiome can lead to increased intestinal permeability, allowing the translocation of bacterial metabolites, toxins, and inflammatory molecules into the systemic circulation. These microbial products, such as LPS and amyloid proteins, can then cross the blood–brain barrier and induce neuroinflammation, oxidative stress, and neuronal dysfunction, hallmarks of Alzheimer’s pathology (**Figure 7**).

The altered gut microbiome in Alzheimer’s disease can contribute to the development and progression of the disease through several mechanisms. These mechanisms illustrate the complex interplay between gut health and neurodegenerative processes in Alzheimer’s disease as follows:

1. Diet-induced gut microbiome dysbiosis: Poor dietary habits alter gut microbial composition, initiating dysbiosis and impairing beneficial metabolite production, such as SCFAs, which are essential for gut and brain health.
2. Gut–blood–brain axis disruption: Dysbiosis affects communication along the gut–brain axis, increasing intestinal permeability (“leaky gut”) and allowing microbial toxins like LPS and bacterial amyloids to translocate into systemic circulation, triggering systemic inflammation (Wu et al., 2021). Changes in the gut microbiome can affect the gut–brain axis, altering communication between the gut and brain, which promotes BBB breakdown, enabling neurotoxic compounds to infiltrate the brain. These processes exacerbate Alzheimer’s disease progression and negatively impact cognitive function and emotional regulation (Cammann et al., 2023).
3. Circulating microbial products: The entry of bacterial toxins to the bloodstream can stimulate immune responses and mimic host proteins, such as Aβ, promoting molecular mimicry and immune activation (Leblhuber et al., 2021).
4. Neuroinflammation via microbial metabolites and immune activation: Circulating microbial products activate systemic immune pathways, inducing the release of pro-inflammatory cytokines such as TNF-α and IL-6, which contribute to tissue damage and prime neuroinflammatory responses (Sait and Day, 2024; Kowalski and Mulak, 2019; Leblhuber et al., 2021). These cytokines engage microglial Toll-like receptors (TLRs), particularly TLR4, triggering chronic neuroinflammation and subsequent neuronal injury.
5. BBB disruption: Pro-inflammatory cytokines, generated from systemic immune activation, compromise BBB integrity through mechanisms involving tight junction protein degradation and increased transcytosis (Sait and Day, 2024). This disruption facilitates the infiltration of peripheral immune mediators, microbial metabolites, and potentially Aβ(Aβ) oligomers into the brain parenchyma, thereby exacerbating neuroinflammation and potentially accelerating Alzheimer’s disease pathology (Kowalski and Mulak, 2019).
6. Metabolite disruption and oxidative stress: The gut microbiome produces metabolites such as SCFAs, which play a role in brain health. An altered microbiome may reduce SCFA production, affecting neuroprotection and energy metabolism in the brain. Dysbiosis reduces SCFA levels (e.g., butyrate), weakening antioxidant defenses and neuroprotection. Simultaneously, harmful metabolites such as trimethylamine N-oxide (TMAO) increase oxidative stress and mitochondrial dysfunction, promoting neuronal injury.
7. Aβ aggregation and cross-seeding: Certain microbial profiles can influence amyloid-β aggregation, a hallmark of AD, thereby accelerating its cerebral deposition. Bacterial amyloids, such as *Escherichia coli* CsgA, share structural similarities with Aβ and promote cross-seeding and misfolding through molecular mimicry, enhancing plaque formation (Savonjie and Weaver, 2023). Additionally, microbial products like LPS and TMAO exacerbate Aβ accumulation by impairing clearance mechanisms, including enzymatic degradation and blood–brain barrier transport. This combined effect facilitates Aβ plaque buildup, contributing to AD pathogenesis (Alicja et al., 2024).
8. Tau hyperphosphorylation: Chronic systemic inflammation and oxidative stress induced by gut microbiome dysbiosis activate tau kinases such as glycogen synthase kinase-3 beta (GSK-3β) and cyclin-dependent kinase 5 (CDK5). This leads to abnormal tau hyperphosphorylation, promoting neurofibrillary tangle (NFT) formation, which disrupts neuronal cytoskeletal integrity and axonal transport (Kowalski and Mulak, 2019; Flynn and Yuan, 2023). Additionally, microbial metabolites like TMAO have been shown to directly exacerbate tau pathology by enhancing kinase activity and oxidative damage (Cammann et al., 2023).
9. Bile acid metabolism disruption: Alterations in gut microbiota composition and function significantly impact bile acid (BA) metabolism. Primary bile acids, synthesized in the liver, are essential for fat digestion and absorption. Dysbiosis leads to a reduction in these primary bile acids and an increase in secondary bile acids, such as deoxycholic acid, produced by bacterial biotransformation of primary BAs. Elevated secondary bile acids have been linked to cognitive decline and the accumulation of Aβ and tau proteins, key pathological features of Alzheimer’s disease (NIH, 2023). Notably, some secondary bile acids can cross the BBB, where they may directly impair neuronal function and exacerbate neurodegenerative processes. Thus, dysbiotic shifts in bile acid profiles contribute to AD pathogenesis by modulating neuroinflammation and neuronal health.
10. Nutrient malabsorption: Gut microbiome dysbiosis impairs the absorption of essential nutrients, including B vitamins (such as B_6_, B_12_, and folate) and trace minerals like zinc, which are vital for neuronal function and cognitive health. Deficiencies in these nutrients exacerbate oxidative stress, impair neurotransmitter synthesis, and contribute to neurodegeneration, thereby worsening cognitive deficits associated with Alzheimer’s disease. Altered microbial communities disrupt nutrient metabolism and intestinal absorption, further compromising brain health and disease progression.
11. Oxidative stress and metabolic dysregulation: Gut microbiome imbalances reduce the production of beneficial SCFAs, such as butyrate, which play a crucial role in protecting neurons against oxidative stress and supporting metabolic homeostasis. The depletion of SCFAs compromises antioxidant defenses and neuronal health, thereby promoting Alzheimer’s disease progression. Conversely, harmful metabolites like TMAO, generated by microbial metabolism of certain dietary nutrients, induce mitochondrial dysfunction and elevate ROS production, accelerating neurodegeneration. The combined impact of amyloid-β plaques, tau pathology, oxidative damage, and nutrient deficiencies culminates in progressive neuronal loss, synaptic dysfunction, and cognitive decline characteristic of AD.
12. Genetic and host–microbiome interactions: Host genetics significantly influence gut microbiome composition and its interaction with immune and metabolic pathways, thereby modulating AD susceptibility and progression. Notably, the APOE ϵ4 allele (Ren et al., 2025) interacts with pro-inflammatory gut microbes such as *Collinsella*, amplifying systemic inflammation and promoting Aβ aggregation. Conversely, APOE variants may also shape microbiome diversity and function, creating a bidirectional relationship. Dysbiotic microbiota associated with genetic risk factors contribute to increased oxidative stress and reduced production of neuroprotective SCFAs like butyrate, weakening antioxidant defenses and neuronal health. Additionally, harmful metabolites such as TMAO, derived from microbial metabolism of certain dietary components, induce mitochondrial dysfunction and ROS generation, accelerating neurodegeneration and AD pathology.

Although the majority of butyrate is consumed locally by colonocytes as a primary energy source, a remaining fraction enters the systemic circulation, crosses the blood–brain barrier, and retains potent neuroprotective actions, making it both a key colonic fuel and a systemically active neuromodulatory metabolite. Butyrate exerts its neuroprotective effects through multiple complementary mechanisms. It is transported into neural cells via monocarboxylate transporters (MCT1), where it activates Nrf2 signaling and upregulates antioxidant enzymes such as superoxide dismutase, catalase, and glutathione peroxidase, thereby limiting reactive oxygen species-mediated neuronal damage (Saikachain et al., 2023). As a histone deacetylase (HDAC) inhibitor, butyrate also promotes the expression of genes involved in neuroprotection, neurogenesis, and mitochondrial stability, including fatty acid binding protein 7 (FABP7/BLBP), which supports mitochondrial function and reduces ROS generation during metabolic stress (Ahmed et al., 2019; Liu et al., 2021).

In parallel, SCFAs attenuate neuroinflammation by dampening microglial TLR4/NF−κB signaling, favoring a shift from pro−inflammatory M1 toward anti−inflammatory M2 phenotypes and lowering the production of ROS−promoting cytokines such as TNF−α and IL−6. They also reinforce gut and blood–brain barrier integrity via upregulation of tight junction proteins, limiting endotoxin translocation and systemic oxidative stress (Aburto and Cryan, 2024). Collectively, adequate SCFA, particularly butyrate, production by a eubiotic microbiota represents a key mechanism through which the gut–brain axis maintains redox balance and neuronal health.

## 7 High-fat diet and Alzheimer’s disease progression

**Figure 8:**
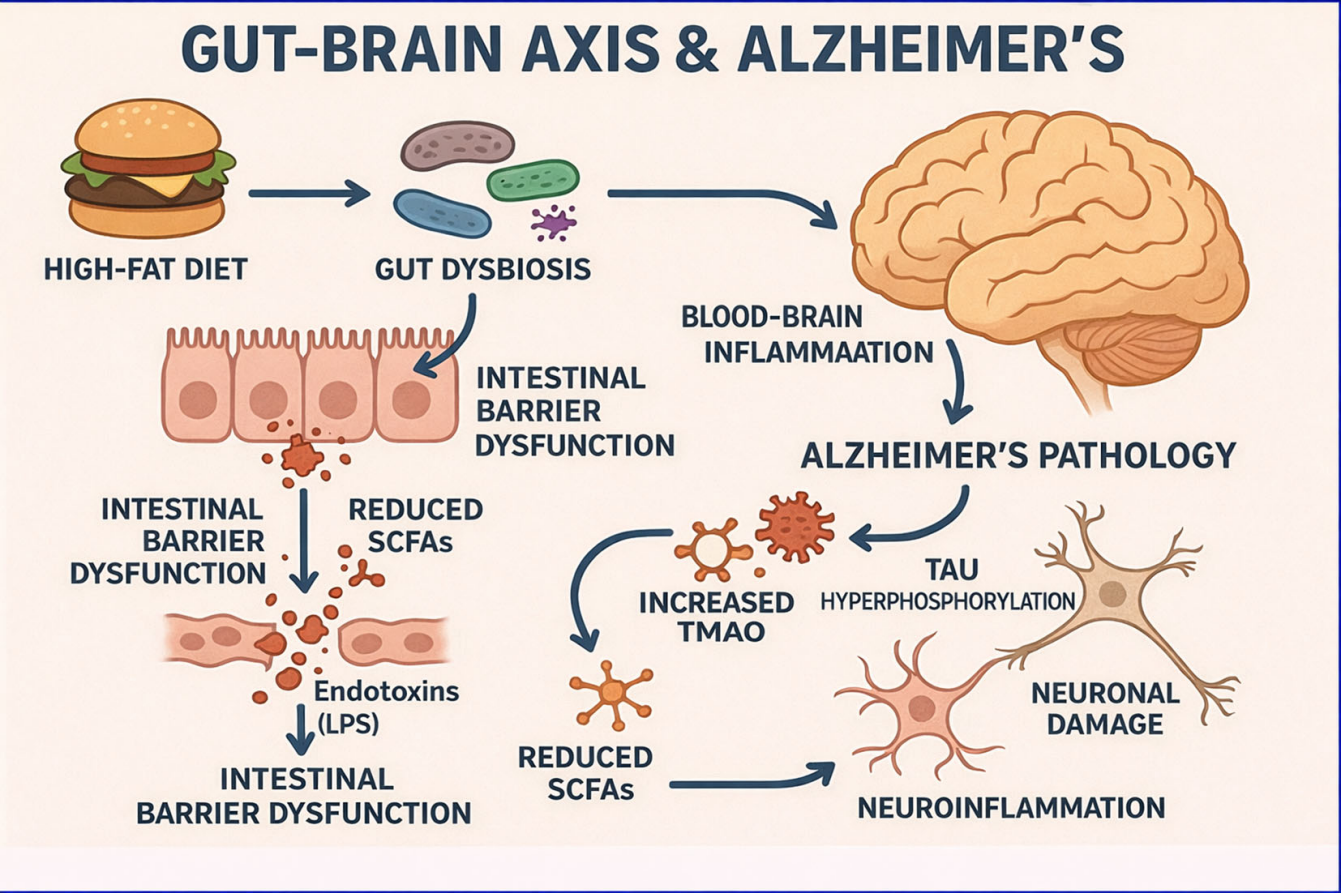
The figure illustrates that a poor diet can contribute to the development of Alzheimer’s disease through the gut–brain axis. It disrupts gut microbiota diversity, leading to intestinal barrier dysfunction and increased production of endotoxins like LPS. These toxins enter the bloodstream, triggering immune dysfunction and systemic inflammation. This, in turn, elevates harmful metabolites such as trimethylamine-oxidized form (TMAO) and activates cytokine signaling, contributing to blood–brain inflammation. As these inflammatory processes reach the brain, they promote tau protein hyperphosphorylation and Aβ plaque buildup, causing neuronal damage, neuroinflammation, and ultimately advancing Alzheimer’s pathology.

Evidence suggests that imbalances in gut microbiota can trigger systemic inflammation, compromise the blood–brain barrier, and modulate amyloid-ß accumulation, hallmarks of Alzheimer’s pathology (**Figure 8**). Multiple interrelated processes are involved in the pathological mechanisms by which a high-fat diet (HFD), especially one rich in saturated fats, contributes to the neurodegeneration, development, and progression of AD. These are discussed below.

### 7.1 Gut microbiome dysbiosis

High-fat diets disrupt the gut microbiota by reducing beneficial bacteria and promoting the growth of pro-inflammatory species. This microbial imbalance shifts the gut environment toward inflammation and toxin production, in which it increases harmful bacteria and decreases beneficial species like *A. muciniphila*, which plays a key role in maintaining gut barrier integrity. Meanwhile, this dysbiosis enhances gut production of endotoxins, bacterial amyloids, and metabolites that contribute to systemic inflammation.

### 7.2 Increased leaky gut

HFD shifts a high diversity microbiome to a low diversity state, enhancing microbial pathogens and leading to degradation of the gut epithelial barrier, thereby increasing intestinal permeability. This function allows harmful substances like LPS, bacterial toxins, and inflammatory mediators to leak into systemic circulation (Nishizawa et al., 2016). This triggers systemic inflammation and primes the immune system for hyperactivation (Sochocka et al., 2019). HFD also influences the production of neuroactive metabolites, including short-chain fatty acids and neurotransmitters, that affect neuronal function and immune responses. These findings offer a compelling hypothesis: The gut microbiome, often overlooked in neurological disorders, might play a pivotal role in driving the pathogenesis of AD.

Pro-inflammatory immune response is characterized by elevated cytokines such as IL-6, TNF-α, and IL-1β. Concurrently, dysbiosis enhances the production of neurotoxic metabolites such as TMAO. These inflammatory mediators and toxic compounds cross the blood–brain barrier, contributing to neuroinflammation, microglial activation, and oxidative stress within the central nervous system. This cascade promotes the hyperphosphorylation of tau proteins and the accumulation of Aβ plaques, the hallmark features of Alzheimer’s pathology, ultimately leading to synaptic dysfunction, neuronal death, and cognitive decline.

### 7.3 Immune system activation and chronic inflammation

The translocation of gut-derived endotoxins activates the innate immune system, especially via TLR4 and TNF signaling pathways.

- LPS–TLR4 pathway activation in the liver systemically promotes chronic “metaflammation” (metabolism-linked inflammation) (Li et al., 2022).
- This inflammation spreads to the brain, as peripheral cytokines cross the BBB, disrupting neuronal function and facilitating neuroinflammation (Rodrigues et al., 2023).

### 7.4 Blood–brain barrier breakdown and brain inflammation

The BBB, much like the intestinal barrier, becomes more permeable under inflammatory stress, allowing inflammatory mediators and microbial by-products to reach the brain.

- This exacerbates microglial activation, neuroinflammation, and the production of neurotoxic substances such as Aβ and tau proteins, which are hallmarks of AD (MacPherson et al., 2023).

### 7.5 Acceleration of Alzheimer’s pathology

Saturated fats can directly activate inflammatory signaling pathways, such as TLR4 and NF-κB. This activation is associated with increased circulating levels of pro-inflammatory cytokines, including IL-1β, IL-6, and TNF-α. These pro-inflammatory cytokines promote tau hyperphosphorylation by upregulating kinases such as GSK-3β and CDK5, facilitating the formation of neurofibrillary tangles.

Elevated levels of cytokines can exacerbate neuroinflammation by crossing the blood–brain barrier or activating endothelial and glial cells to produce secondary inflammatory mediators. Activated microglia produce more amyloid-β and impair its clearance. Longitudinal studies show that elevated inflammatory markers (CRP, IL-6) are associated with increased risk of cognitive decline and Alzheimer’s disease. Anti-inflammatory interventions show promise in slowing AD progression. Inflammation and microbial metabolites (e.g., bacterial amyloids) can mimic and promote misfolded protein aggregation in the brain, promoting AD development. Meanwhile, dysbiotic bacteria produce amyloid-like proteins that may cross-seed human Aβ, exacerbating plaque formation (Tarawneh and Penhos, 2022). SCFA depletion (e.g., butyrate), which is linked to beneficial anti-inflammatory effects in the brain, is commonly seen in HFD models and AD (Mo et al., 2024), disrupting brain glucose metabolism and nutrient transport. Blood–brain barrier disruption is also detected.

How a high-fat diet causes it:

- Circulating LPS and inflammatory cytokines damage BBB tight junctions.
- Reduced SCFA production impairs BBB maintenance.
- Oxidative stress from high-fat metabolism damages BBB endothelial cells.

In general, high-fat diets initiate gut microbiome dysbiosis, leading to increased intestinal and blood–brain barrier permeability, systemic immune activation, and neuroinflammation, ultimately accelerating Alzheimer’s pathology. High-fat diets, especially those rich in saturated and trans fats, promote gut dysbiosis characterized by increased Firmicutes/Bacteroidetes ratio and reduced beneficial bacteria like *A. muciniphila*.

Contribution of HFD to Alzheimer’s disease:

HFD contributes to the enhancement and formation of the following:

- TMAO promotes neuroinflammation and amyloid aggregation. Metabolomic studies show distinct patterns in AD patients, with elevated TMAO levels correlating with cognitive impairment severity. Microbiome transplantation from AD patients to germ-free mice transfers cognitive deficits, suggesting causative metabolite effects, and increases the production of harmful metabolites (TMA/TMAO, secondary bile acids).
- Reduced tryptophan metabolites affect serotonin and kynurenine pathways.
- Altered bile acid metabolism affects brain cholesterol homeostasis.
- The production of beneficial neurotransmitter precursors is decreased.

## 8 Western-style diet

Studies have shown that a Western-style diet, characterized by high intake of processed foods, sugar, and saturated fat, is associated with an increased risk of cognitive decline and Alzheimer’s disease. In contrast, a diet rich in fruits, vegetables, whole grains, and lean protein sources may help promote a healthy gut microbiome and reduce the risk of cognitive decline. By making informed dietary choices, individuals may be able to reduce their risk of developing Alzheimer’s disease and promote overall brain health. The Western-style diet is defined by a high intake of saturated fats, refined sugars, processed foods, red and processed meats, and sugary beverages, alongside a low consumption of fruits, vegetables, whole grains, nuts, and dietary fiber. This dietary pattern is prevalent in many industrialized nations and has become increasingly common worldwide due to the widespread availability of ultra-processed foods and shifts in food production and consumption habits. Over the long term, adherence to a Western diet has profound consequences for human health. The Western diet’s departure from traditional nutritional models has led to various health challenges, including obesity, diabetes, heart disease, and cancer, largely through mechanisms involving chronic low-grade inflammation, metabolic dysfunction, and oxidative stress.

**Figure 9:**
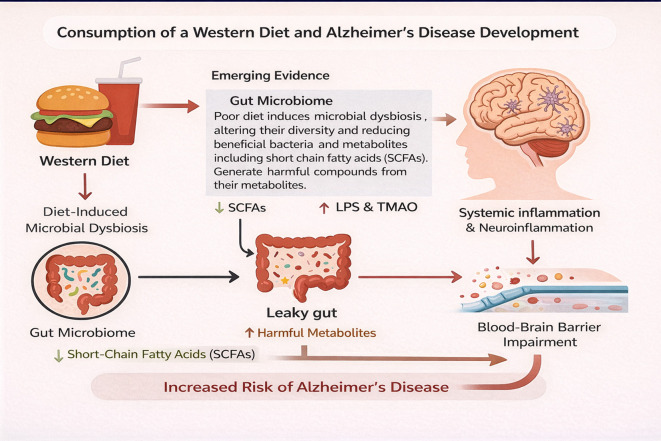
Consumption of a Western diet and Alzheimer’s disease development. This diagram illustrates how a Western diet can induce microbial dysbiosis, altering microbial diversity and resulting in a reduction of beneficial microbiota. This disruption leads to increased intestinal permeability (leaky gut), which decreases the production of short-chain fatty acids and promotes the generation of harmful metabolites. These metabolites can cross the blood–brain barrier, collectively increasing the risk of Alzheimer’s disease development.

Emerging evidence suggests that one of the key mechanisms linking the Western diet to AD is through its detrimental impact on the gut microbiome. Diet-induced microbial dysbiosis alters the composition and metabolic function of gut bacteria, reducing the production of beneficial metabolites such as SCFAs and increasing the generation of harmful compounds like LPS and TMAO. These changes compromise intestinal barrier integrity, leading to a “leaky gut” that allows bacterial products to enter systemic circulation. The resulting systemic inflammation and immune activation further impair the BBB, facilitating the entry of neurotoxic substances into the brain (**Figure 9**).

Within the central nervous system, these inflammatory processes activate microglia and astrocytes, shifting them toward a pro-inflammatory phenotype. Chronic neuroinflammation contributes to a hallmark of AD pathologies, including the accumulation of misfolded Aβ plaques and tau hyperphosphorylation, which lead to the formation of neurofibrillary tangles. Additionally, oxidative stress induced by mitochondrial dysfunction and pro-oxidant metabolites accelerates neuronal damage and synaptic loss.

The Western diet also contributes to deficits in essential nutrients, such as B vitamins, omega-3 fatty acids, antioxidants, and polyphenols, which are crucial for maintaining neuronal health and preventing cognitive decline. In contrast, adherence to a diet rich in whole, unprocessed foods, such as the Mediterranean or DASH diet, has been associated with a more diverse and balanced gut microbiota, improved metabolic outcomes, and reduced risk of cognitive impairment and AD.

In summary, the Western-style diet promotes a cascade of pathophysiological events—gut dysbiosis, systemic inflammation, BBB disruption, neuroinflammation, and protein misfolding—that converge to drive the onset and progression of Alzheimer’s disease. Adopting healthier dietary patterns may offer a promising preventive strategy to maintain cognitive function and reduce AD risk.

## 9 High-sugar consumption

The oral microbiota occupies an extremely important position within the human microbiome and is the second-largest microbiota in humans after that in the gut (Fak et al., 2015). There is new scientific evidence recently published that aside from the gut microbiome, oral microflora is also able to influence brain functions (Orr et al., 2020). Numerous studies have shown that periodontal disease is associated with neurodegeneration and cognitive decline (Kamer et al., 2008; Cerajewska et al., 2016; Wang et al., 2019). In a mouse study, long-term consumption of a high-sugar diet altered gut microbiota composition, increasing harmful Firmicutes and decreasing beneficial Bacteroidetes, which is associated with gut barrier disruption (Alasmar et al., 2023). Another study using rats fed high-fructose diets showed increased gut permeability, endotoxemia, and hepatic lipid accumulation, linking sugar intake to intestinal barrier dysfunction and systemic inflammation (Ochoa-Repáraz and Kasper, 2014). In humans, excessive sugar intake promotes microbial dysbiosis in the oral microbiome by lowering pH and providing fermentable substrates that favor acidogenic and pathogenic bacteria such as *Porphyromonas gingivalis* and *Streptococcus* species. This leads to reduced microbial diversity and an altered biofilm structure conducive to periodontal inflammation. Dysbiosis in the gut similarly disrupts barrier integrity, increasing intestinal permeability (leaky gut) and allowing endotoxins to enter circulation while reducing beneficial SCFA-producing microbes. This microbial disruption drives chronic systemic inflammation, which may exacerbate neuroinflammatory and metabolic conditions.

The paths through which oral and intestinal microbiota enter the CNS include translocation via the bloodstream and lymphatic system following increased intestinal permeability, direct neural pathways such as the vagus nerve, and disruption of the BBB through inflammatory mediators. These routes allow microbial components and metabolites to trigger neuroinflammation and modulate CNS function, forming the basis of the oral–gut–brain axis communication network.

**Figure 10:**
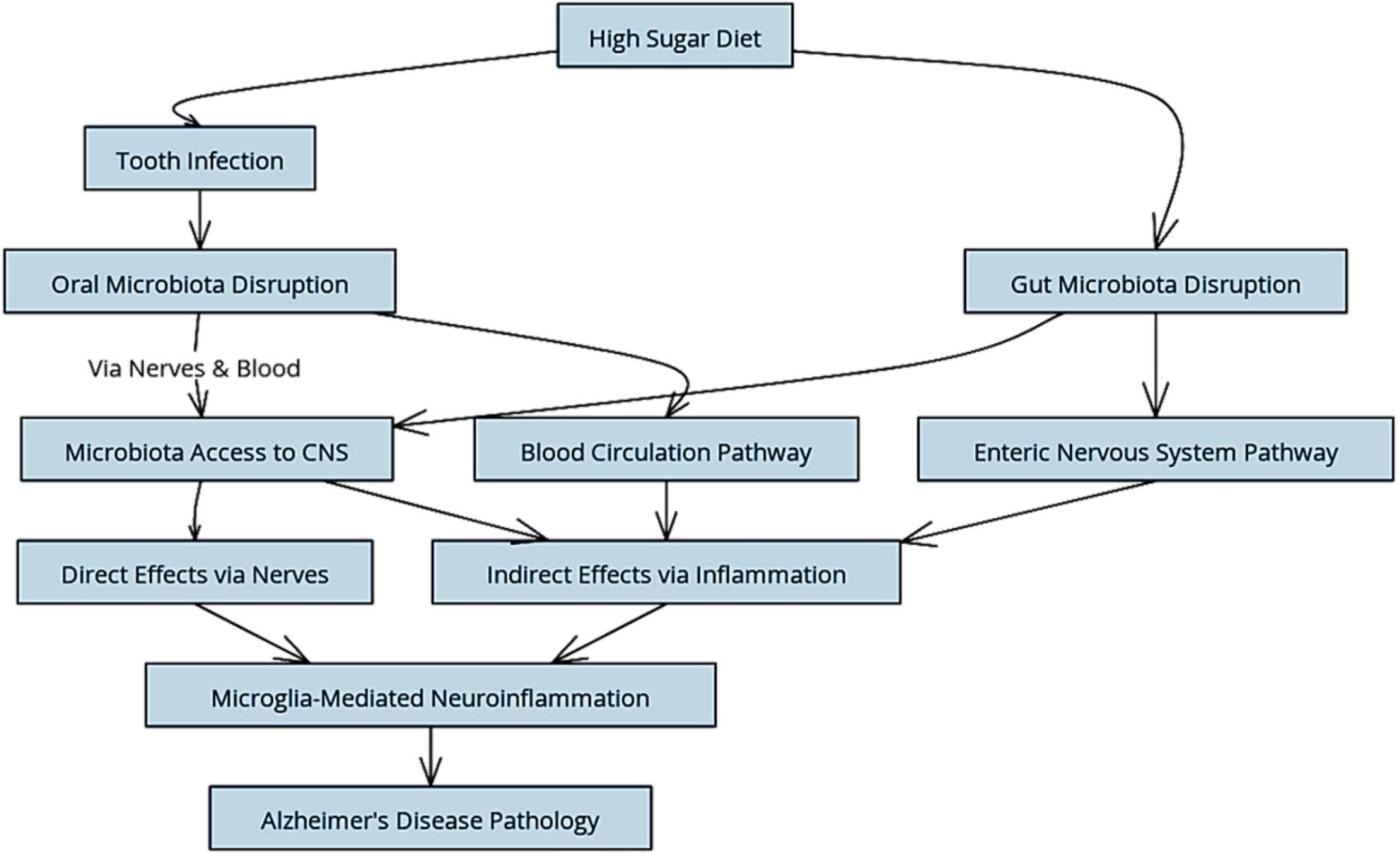
Consumption of high-sugar content diet and Alzheimer’s disease development. This diagram illustrates how a high-sugar diet can initiate a cascade leading to Alzheimer’s disease in which excessive sugar intake promotes tooth infection, disrupting the oral microbiota, which can access the brain via cranial nerves or the bloodstream. Simultaneously, it disrupts the gut microbiota, influencing the brain through the enteric nervous system and systemic inflammation. Both pathways, oral and gut, converge in the central nervous system, triggering microglia-mediated neuroinflammation, a key contributor to Alzheimer’s pathology.

Oral bacterial species and their products can affect the brain either directly through neural pathways such as the trigeminal, olfactory, and facial nerves, as well as via the circulating bloodstream, or indirectly through disturbances in the gut microbiota that promote systemic inflammation and neuroinflammatory responses (**Figure 10**). Meanwhile, gut microbiota and their products affected the brain either directly through the enteric nervous system or indirectly through mediating systemic inflammation. Both direct and indirect effects from oral and intestinal microbiota contributed to microglia-mediated neuroinflammation, resulting in AD-related pathologies.

## 10 Microorganisms associated with Alzheimer’s disease

Emerging research has identified several microorganisms that may play a role in the development and progression of AD. Among them, oral pathogens such as *P. gingivalis* (Huang et al., 2025b), a key bacterium in periodontal disease, have been found in the brains of AD patients, suggesting a potential link between chronic oral infection and neurodegeneration. *Fusobacterium* sp. Also has well-documented roles in dysbiosis and systemic inflammation. This bacterium, particularly *Fusobacterium nucleatum*, is a well-documented contributor to microbial dysbiosis and systemic inflammation. It promotes inflammation by disrupting the intestinal epithelial barrier, inducing pro-inflammatory cytokines, such as IL-1β, IL-6, IL-17F, and TNF-α, and activating signaling pathways including NF-κB and the IL-17F axis (Zheng et al., 2025). Additionally, it modulates immune responses by promoting macrophage polarization toward the pro-inflammatory M1 phenotype, further compromising mucosal barrier integrity (Li et al., 2025).

Moreover, important pathogenic factors include LPS, gingipains, and TMAO. Additionally, herpes simplex virus type 1 (HSV-1), a common neurotropic virus, has been implicated in AD due to its ability to establish latent infections in the nervous system and reactivate under stress or immunosuppression. Certain strains of gut bacteria, including *E. coli* and *B. fragilis*, may also contribute to cognitive decline by influencing systemic inflammation and producing neurotoxic metabolites. These microorganisms, through Various direct and indirect pathways can trigger neuroinflammation, promote Aβ(Aβ) accumulation, and impair neuronal function, key processes involved in the pathogenesis of AD. Several microbial species, originating from both the oral cavity and the gut, have been identified in brain tissue or cerebrospinal fluid of patients with AD. For instance, *P. gingivalis*, a predominant periodontal pathogen, has been detected in the hippocampal and cortical regions of AD patients, correlating with classic AD-like pathology such as neuroinflammation, neurodegeneration, tau phosphorylation, and the formation of both intracellular and extracellular Aβ plaques and neurofibrillary tangles. Similarly, *Treponema denticola*, another oral spirochete, has been found in higher concentrations in the brain tissues of AD patients compared to healthy controls, suggesting a potential association between spirochetes and cognitive impairment (Li et al., 2024).

In addition to oral microbiota, gut microbiota dysbiosis has been implicated in AD pathogenesis. Alterations in the gut microbiota are associated with elevated levels of inflammatory cytokines, oxidative stress, and neurotoxicity in the brains of both AD patients and animal models. Gut microbes release a range of neuromodulatory metabolites, including SCFAs, which can cross the BBB and influence brain function. However, enteric dysbiosis leads to attenuated SCFA generation, resulting in neurological dysfunction and disease (Yang et al., 2024). Several microbial species, originating from both the oral cavity and the gut, have been identified in the brain tissue or cerebrospinal fluid of patients with AD (**Tables 1**, **2**). These microorganisms are increasingly recognized as potential contributors to disease progression by disrupting immune homeostasis, crossing the blood–brain barrier, and influencing the gut–brain axis.

**Table 1:** Microorganisms associated with Alzheimer’s disease.

Microorganism	Main colonization	References	Role in Alzheimer’s disease development
*Candida albicans*	Oral cavity, intestine	Bulgart et al. (2020)	Can trigger systemic inflammation and produce neurotoxic metabolites contributing to neurodegeneration.
*Escherichia coli*	Intestine	Zhan et al. (2016)	Some strains release endotoxins (LPS) that may disrupt the BBB and promote neuroinflammation linked to AD pathology.
*Staphylococcus epidermidis*	Intestine	Zhao et al. (2017)	Generally commensal but can modulate immune responses that may indirectly affect brain inflammation.
*Staphylococcus aureus*	Intestine	Bulgart et al. (2020)	Produces exotoxins provoking systemic inflammation, potentially exacerbating neurodegeneration processes.
*Listeria monocytogenes*	Intestine	Bulgart et al. (2020)	Known for CNS invasion in infections; may contribute to neuroinflammatory responses relevant to AD progression.
*Enterococcus faecalis*	Oral cavity/intestine	Bulgart et al. (2020)	Can produce inflammatory mediators influencing systemic inflammation linked to neurodegenerative disease development.
*Pseudomonas aeruginosa*	Intestine	Bulgart et al. (2020)	Produces endotoxins and virulence factors that may exacerbate systemic inflammation affecting the CNS.
*Helicobacter pylori*	Intestine	Zhan et al. (2016)	Infection associated with an increased risk of cognitive decline; triggers systemic inflammation and amyloid accumulation.
*Bacteroides fragilis*	Intestine	Zhao et al. (2017)	Its toxins may disrupt the gut barrier, facilitating neuroinflammation linked to AD.
*Streptococcus mitis*	Oral cavity	Bulgart et al. (2020)	Can contribute to oral dysbiosis and inflammation, indirectly influencing neuroinflammatory pathways.
*Streptococcus salivarius*	Oral cavity	Bulgart et al. (2020)	Generally beneficial but imbalances may contribute to inflammation affecting the CNS.
*Porphyromonas gingivalis*	Oral cavity	Liu et al. (2019)	A key periodontal pathogen; produces gingipains and LPS implicated in AD by promoting neuroinflammation and amyloidogenesis.
*Actinobacillus actinomycetemcomitans*	Oral cavity	Wan and Fan (2023)	Periodontal pathogen promoting systemic inflammation impacting neurodegeneration.
*Tannerella forsythia*	Oral cavity	Wan and Fan (2023)	Another periodontal pathogen involved in inflammatory cascades contributing to AD progression.
*Treponema denticola*	Oral cavity	Wan and Fan (2023)	Spirochete implicated in neurodegenerative mechanisms through chronic inflammation.
*Streptococcus mutans*	Oral cavity	Wan and Fan (2023)	Associated with dental caries and oral inflammation, potentially influencing systemic inflammation.
*Fusobacterium nucleatum*	Oral cavity	Wan and Fan (2023)	Contributes to oral and gut dysbiosis; produces inflammatory mediators linked to neuroinflammation in AD.
*Actinomyces meyeri*	Oral cavity	Wan and Fan (2023)	Involved in oral infections and inflammation, potentially affecting systemic immune responses relevant to AD.

**Table 2:** Viruses associated with Alzheimer’s disease (AD).

Microorganism	Main colonization	References	Role in Alzheimer’s disease development
*Herpesviruses*	Oral cavity	Zhan et al. (2016)	Can cause latent infections and reactivation, triggering neuroinflammation, linked to AD pathology.
*Cytomegalovirus*	Oral cavity	Zhan et al. (2016)	Associated with neuroinflammation and cognitive decline in AD.

## 11 Future concept

Future research on diet and AD should focus on elucidating the precise molecular and cellular mechanisms through which dietary patterns influence neurodegeneration. This includes deeper investigation into how specific nutrients and bioactive compounds modulate neuroinflammation, amyloid-beta accumulation, tau pathology, oxidative stress, and mitochondrial function in the aging brain. There is growing interest in the role of the gut microbiome as a mediator of diet’s effects on brain health, including how diet-induced microbial shifts affect gut barrier integrity, systemic inflammation, and the microbiota–gut–brain axis in AD progression.

## 12 Conclusion

The lack of causal treatment for Alzheimer’s disease is mainly due to the unknown disease etiology. Currently, there are several hypotheses regarding the etiology of Alzheimer’s disease, e.g., amyloid, ischemic, or hygiene theory, which attempt to explain the mechanism of development of Alzheimer’s disease, but none of these hypotheses fully explain the disease’s origin. Currently, the causative agents of this disease are based on many universally known mechanisms of neurodegeneration, including dysregulation of calcium homeostasis, abnormal accumulation of amyloid and dysfunctional tau protein, imbalance of neurotransmitters, necrotic and apoptotic neuronal death, disappearance of synapses, neuroinflammation with pathological microglia and astrocyte activation in the brain, white matter changes, and finally, brain atrophy. Recent studies strongly suggest that gut and oral microbiome are capable of modulating the neurochemical and neurometabolic signaling pathways of the brain through the formation of a two-way communication axis involving the endocrine and immune systems and contribute to the development of neuron inflammation and neurodegeneration. In turn, there is a strong correlation that exists between AD and the type of diet and food ingredients. Consequently, oral- and intestinal-specific bacterial species and their products, considered potential biomarkers for the prevention and clinical diagnosis of AD, can be modulated by a healthy diet and probiotic bacteria. Emerging evidence highlights the significant role of diet in modulating these pathological processes, particularly through its impact on the gut microbiome and systemic inflammation. High-fat, Western-style diets, characterized by high saturated fats, refined sugars, and low fiber, have been associated with gut dysbiosis, increased neuroinflammation, and exacerbation of Alzheimer’s pathology, including Aβ aggregation and tau hyperphosphorylation. Conversely, dietary patterns such as the Mediterranean diet, rich in antioxidants, polyunsaturated fatty acids, and anti-inflammatory components, have demonstrated protective effects against cognitive decline and neurodegeneration, potentially by preserving gut microbiota balance and reducing systemic and neuroinflammation. Therefore, understanding the influence of specific dietary patterns on neuroimmune mechanisms and gut–brain axis communication is critical for developing preventive and therapeutic strategies targeting Alzheimer’s disease. Integrating dietary interventions with modulation of the gut microbiome represents a promising avenue for mitigating disease progression and improving cognitive outcomes in at-risk populations.
